# Application of Amorphous Zirconium-Yttrium-Aluminum-Magnesium-Oxide Thin Film with a High Relative Dielectric Constant Prepared by Spin-Coating

**DOI:** 10.3390/membranes11080608

**Published:** 2021-08-10

**Authors:** Huiyun Yang, Zhihao Liang, Xiao Fu, Zhuohui Xu, Honglong Ning, Xianzhe Liu, Jiajing Lin, Yaru Pan, Rihui Yao, Junbiao Peng

**Affiliations:** 1 State Key Laboratory of Luminescent Materials and Devices, Institute of Polymer Optoelectronic Materials and Devices, South China University of Technology, Guangzhou 510640, China; 201830320362@mail.scut.edu.cn (H.Y.); 201530291443@mail.scut.edu.cn (Z.L.); 201630343721@mail.scut.edu.cn (X.F.); 201964172349@mail.scut.edu.cn (J.L.); 201930173424@mail.scut.edu.cn (Y.P.); psjbpeng@scut.edu.cn (J.P.); 2 Guangxi Key Lab of Agricultural Resources Chemistry and Biotechnology, Yulin Normal University, Yulin 537000, China; xzh21@ylu.edu.cn; 3 Research Center of Flexible Sensing Materials and Devices, School of Applied Physics and Materials, Wuyi University, Jiangmen 529020, China; msliuxianzhe@mail.scut.edu.cn

**Keywords:** zirconium-yttrium-aluminum-magnesium-oxide, dielectric layer, amorphous metal oxide, thin film, solution method

## Abstract

Amorphous metal oxide has been a popular choice for thin film material in recent years due to its high uniformity. The dielectric layer is one of the core materials of the thin film transistor (TFT), and it affects the ability of charges storage in TFT. There is a conflict between a high relative dielectric constant and a wide band gap, so we solved this problem by using multiple metals to increase the entropy of the system. In this paper, we prepared zirconium-yttrium-aluminum-magnesium-oxide (ZYAMO) dielectric layers with a high relative dielectric constant using the solution method. The basic properties of ZYAMO films were measured by an atomic force microscope (AFM), an ultraviolet-visible spectrophotometer (UV-VIS), etc. It was observed that ZYAMO thin films had a larger optical band when the annealing temperature increased. Then, metal-insulator-metal (MIM) devices were fabricated to measure the electrical properties. We found that the leakage current density of the device is relatively lower and the ZYAMO thin film had a higher relative dielectric constant as the concentration went up. Finally, it reached a high relative dielectric constant of 56.09, while the leakage current density was no higher than 1.63 × 10^−6^ A/cm^2^@ 0.5 MV/cm at 1.0 M and 400 °C. Therefore, the amorphous ZYAMO thin films has a great application in the field of high permittivity request devices in the future.

## 1. Introduction

The research on insulating layers is a heated topic in the field of thin film transistors (TFTs), since gate dielectric materials have great effects on the device as a key component of TFT [1,2,3,4,5], and more concern about better performance of the gate dielectric materials has been attracted nowadays. Dielectric properties of materials determine the ability to store charges and influence the leakage current between gate and active layer directly. Due to the exceptional permittivity, high uniformity, good transmittance and large capacitance, amorphous metal oxide semiconductors have attracted wide concern in recent years [6,7,8,9,10]. Metal oxide semiconductor thin films can be prepared by traditional methods [11,12,13], but those are of high costs and slow processing speed [14]. By contrast, the solution method is cheaper, the chemical composition is easier to regulate and the obtained thin film is more uniform and smoother [15,16,17,18].

The oxides applied to the insulating layer have been a single-metal or binary-metal system so far, for example, AlO_x_ [19,20], HfO_x_ [21], YO_x_ [9,22], AlZrO_x_ [23,24], YAlO_x_ [25], LaZrO_x_ [26], ZnMgO_x_ [27], etc. However, most of them can only make one breakthrough in the band gap, leakage current or permittivity. Zirconia is a highly polarizable material with an outstanding relative dielectric constant (~27) [23,28,29]. A higher relative dielectric constant enables a higher capacitance density, thereby reducing the tunneling current of gate dielectric layers and operating voltage of TFT [30,31]. Aluminum oxides have the advantages of a wide band gap and high crystallization temperature [23,32], compensating for the shortcomings of easy crystallization, low breakdown current and high leakage current of zirconia [9,31]. The research results from Ku and Chen et al. [33,34] showed that Mg^2+^ can suppress oxygen vacancy defects because of its high oxygen affinity. This is an effective way to solve the high leakage current caused by defects [5,23,35]. Y^3+^ is a good carrier inhibitor due to the low electronegativity and small standard electrode potential of yttrium, as shown in the studies done in 2012 and 2014 [36,37]. In addition, ZrO_2_, Y_2_O_3_, and MgO have cubic crystal structures, while Al_2_O_3_ has an amorphous structure. Similarity in structure helps to maintain the physical properties, and an amorphous structure is beneficial to improve the interfacial properties.

The superiority of different oxides or ions is beneficial to optimize the properties of the film, and the multi-component oxide dielectric is a feasible system to achieve this goal. In the study, we found that a possible way to solve the conflict between high relative dielectric and a wide band gap is to regulate the entropy of the system [38]. Therefore, we combined four metal oxides to achieve a high relative dielectric constant and a large band gap by increasing the entropy of the system, and the quaternary-metal system was prepared by mixing alumina, zirconia, yttria, and magnesia in this study. Moreover, the novel system also conserves energy, has low costs and is environmentally friendly. The ZYAMO thin films can be applied in thin film transistors, supercapacitor, wearable devices, flexible display, etc., since the produced layers are amorphous and with a high relative dielectric constant.

## 2. Materials and Methods

Five concentrations (0.2 M/0.4 M/0.6 M/0.8 M/1.0 M) of zirconium-yttrium-aluminum-magnesium-oxide (ZYAMO) precursors were prepared by dissolving zirconium nitrate pentahydrate (Zr(NO_3_)_4_·5H_2_O), yttrium nitrate hexahydrate (Y(NO_3_)_3_·6H_2_O), aluminum nitrate nonahydrate (Al(NO_3_)_3_·9H_2_O), and magnesium acetate tetrahydrate (C_4_H_6_MgO_4_·4H_2_O) in ethylene glycol monomethylether (EGME). The molar ratio of the four metals is 1:1:1:1. These solutions were stirred for 8 h, and then aged for 24 h.

**Figure 1:**
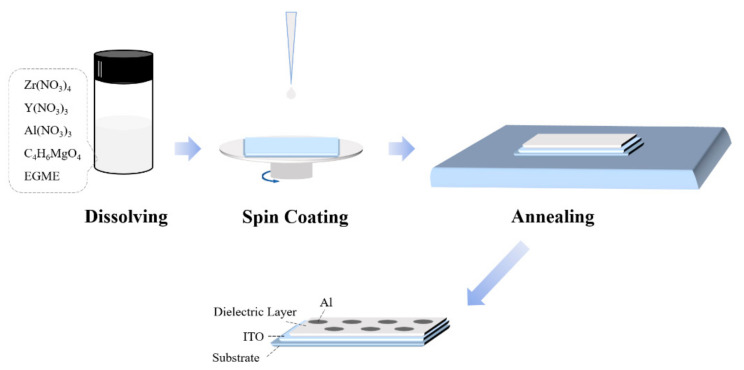
Sketch map of the device fabrication process.

As Figure 1 shows, the mixed precursors were spin-coated on the glass substrate at 5000 r/min for 30 s to obtain thin films. After repeating spinning and pre-annealing at 130 °C for 10 min three times, annealing was carried out at 300 °C to 500 °C for 1.5 h in the air.

Under the same conditions, the ZYAMO dielectric layer was also spin-coated on the glass substrate deposited ITO, and then the Al electrodes were deposited on the gate dielectrics. Thus, the metal-insulator-metal (MIM) devices were finished.

As for the contact angle measurements, we dropped the precursors directly on the glass substrate without annealing; then, the contact angles were tested by an Attension Theta Lite (TL200, BiolinScientific, Gothenburg, Sweden), the surface features of ZYAMO films were observed by a laser scanning confocal microscopy (LSCM, OLS50-CB, Tokyo, Japan), the thicknesses of the dielectric layer were measured by a step profiler (Dektak 150, Veeco, Tucson America), the functional groups in the dielectric material were studied by a Fourier transform infrared spectroscopy (FTIR, ATR Accessory, Nexus, Madison USA), the morphological characteristics of the insulating films were observed by an atomic force microscope (AFM, BY3000, Being Nano-Instruments, Guangzhou, China), an ultraviolet-visible spectrophotometer (UV-VIS, UV-3600SHIMADZU, Kyoto, Japan) was used to analyze the optical properties of films, a thermogravimetric (TG) analyzer was used to measure the thermal behaviors of the precursor solution at a heating rate of 10 °C/min from room temperature to 600 °C, and an X-ray photoelectron spectroscopy (XPS) (Thermo Fisher Scientific, Waltham, MA, USA) analysis was carried out to investigate the chemical composition of the ZYAMO thin films, with the carbon 1 s peak (284.8 eV) as a calibration reference.

The current–voltage (I-V) and capacitance–voltage (C-V) characteristics of the MIM device were measured by the Keithley 4200 (Tektronix, Beaverton, OR, USA) parameter analyzer under ambient conditions.

## 3. Results and Discussion

### 3.1. Surface Properties and Optical Properties

**Figure 2:**
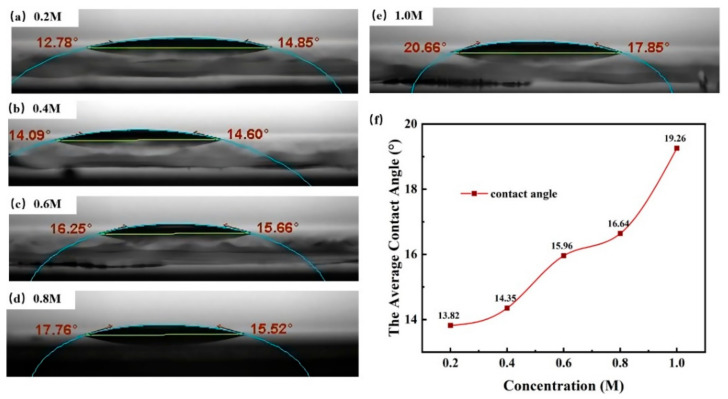
Surface contact angle of different concentrations solutions: (**a**) 0.2 M, (**b**) 0.4 M, (**c**) 0.6 M, (**d**) 0.8 M, (**e**), and 1.0 M. (**f**) Relationship between concentrations and the average contact angle.

Precursors from low concentration (0.2 M) to high concentration (1.0 M) are all clear, colorless, and transparent, exhibiting a good stability. As shown in Figure 2, the contact angle of the precursors still remained relatively low at a high concentration. The good wettability ensured a successful progress of the spin coating preparation and device fabrication.

**Figure 3:**
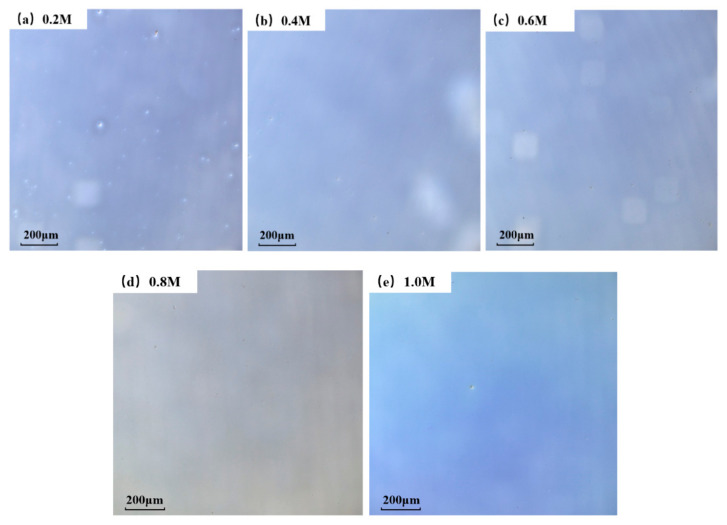
Optical microscopy of ZYAMO thin films annealed at 300 °C at different concentrations: (**a**) 0.2 M, (**b**) 0.4 M, (**c**) 0.6 M, (**d**) 0.8 M, and (**e**) 1.0 M.

A laser scanning confocal microscopy (LSCM) was used to test the morphological characteristics, and the microphotographs of 300 °C group are displayed in Figure 3. Similar trends also appeared in 400 °C and 500 °C conditions. It can be seen from the microphotographs that the density of white particles on the surface decreases as the concentration rises. We speculated that this phenomenon is caused by a faster reaction rate under higher a concentration condition. For a chemical reaction (1), it can be calculated with Equation (2) that the reaction rate γ is bigger when the concentration of reactants increases [39].

mA + n→BC(1)

(2) $$\gamma =K\cdot {\left[A\right]}^{x}\cdot {\left[B\right]}^{y}$$

where ***K*** is the reaction rate constant, [***A***] and [***B***] are the concentration of reactants, and ***x*** and ***y*** are the reaction order. Therefore, the dissolution of the metal salts accelerated, the contact area between solute and solvent increased, and then the formation of metal hydroxides and metal oxides was faster as the concentration went up. Therefore, the dissolution of the metal salts accelerated, the contact area between solute and solvent increased, and the formation of metal hydroxides and metal oxides was faster as the concentration went up.

**Figure 4:**
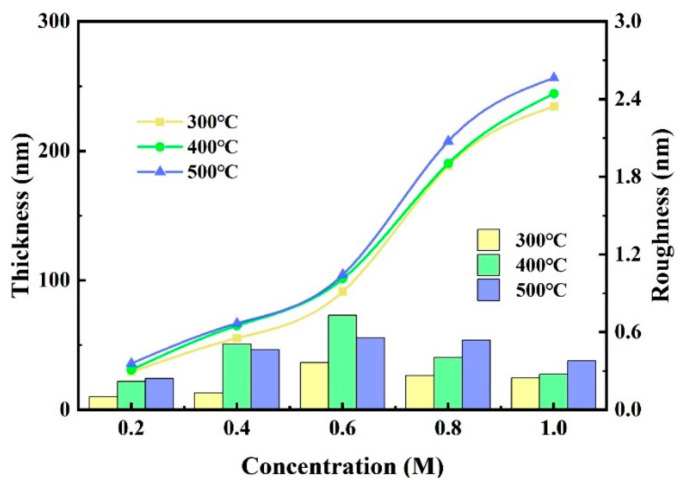
ZYAMO thin film thickness and roughness under different annealing temperatures and solution concentrations.

**Figure 5:**
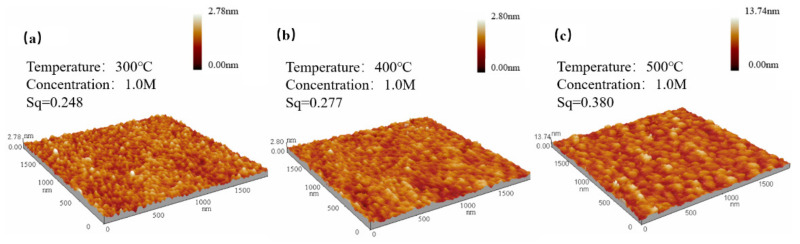
ZYAMO thin film AFM images of 1.0 M after different temperature annealing: (**a**) 300 °C, (**b**) 400 °C, and (**c**) 500 °C.

We measured the thickness and the roughness through a step profiler and an atomic force microscope (AFM), and the results are shown in Figure 4 and Figure 5. The thicknesses of ZYAMO thin films increased rapidly when the concentration rose from 0.2 M to 1.0 M, as shown in Figure 4. Moreover, the thickness increased when the temperature went up. This is probably the result of the change of nucleation condition [40,41]. The critical condition of free energy of nucleation and the barriers are smaller at the initial stage when the temperature is lower, so it is beneficial to form a fine and continuous film at lower temperatures. When the temperature climbs up, a coarser island-shaped organization is formed [9]. By contrast, there is a difference of roughness in the 0.4 M and 0.6 M groups. The roughness of thin film annealed at 400 °C is larger compared with the other two temperatures, possibly owing to the influence of the ligand. The formation of complexes was faster when the temperature reached 500 °C under the condition of 0.4 M and 0.6 M. The complexes evaporated quickly from the surface under these conditions, leaving the atoms in the system to form a metal-oxygen-metal compact structure [22].

From Figure 5, it can be observed that the root mean square increased since the trend of crystallization is more obvious when raising up the temperature, which is consistent with the results in Figure 4.

**Figure 6:**
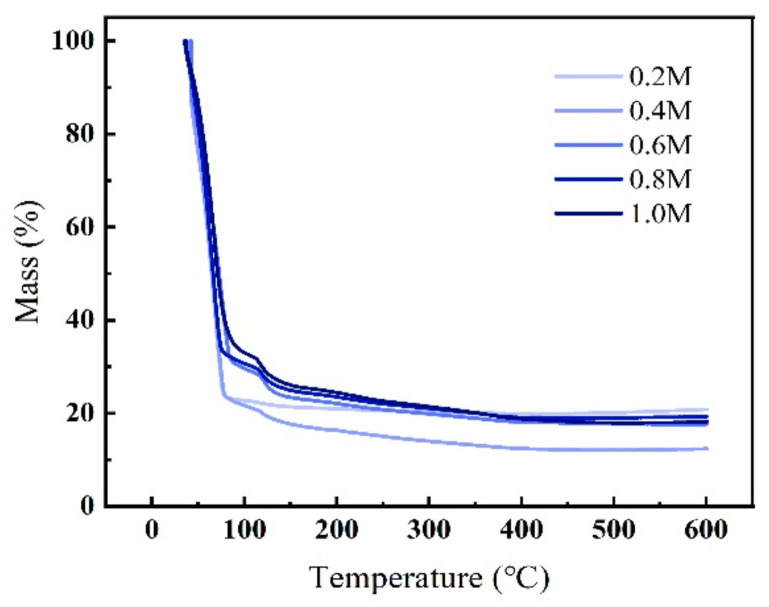
Thermal behavior of the ZYAMO precursor solution analyzed by the thermalgravimetric (TG) analyzer.

In order to confirm the chemical reactions that may occur in the system and analyze the effects of the products on the properties of the film, we performed a test through a thermalgravimetric (TG) analyzer, and the results are shown in Figure 6. There is an obvious mass loss from 25 °C to 75 °C, and we inferred that this is probably because of the quick evaporation of the solvent, as shown in previous studies [42,43,44,45]. The boiling point of EGME is around 125 °C, but the temperature fell in the test, which may be caused by the multiple solutes in the system. Moreover, another mass loss can be observed in the range of 110 °C to 120 °C, and this change is possibly attributed to the decomposition of the nitrates and acetate [46,47,48].

**Figure 7:**
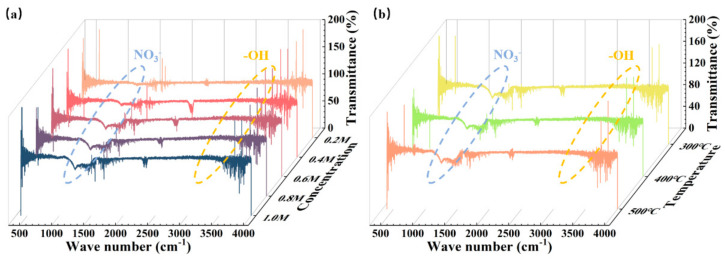
ZYAMO thin film FTIR results: (**a**) 300 °C annealing temperatures and (**b**) 0.25 M solution concentrations.

Apart from that, we also took tests through a Fourier transform infrared spectrometer (FTIR) and the obtained results are shown in Figure 7. The infrared spectroscopy ranges [26,49,50] of hydroxyl and nitrate are 3000–3500 cm^−1^ and 1200–1500 cm^−1^, so the hydroxyl group and the nitrates can be substantially removed after annealing from the graph results. The amount of nitrate left in the film had a slight rise when the precursors’ concentration went up, but the annealing temperature had little effect on these relevant groups.

We surmised that the possible reactions may occur in the system are hydrolysis, condensation, dehydroxylation, oxygen binding, etc., based on the above results and other studies [51,52,53]. The possibly reaction Equations (3)–(8) are listed as follows:

M_1_(NO_3_)_X_·nH_2_O+ M_2_(NO_3_)_X_·nH_2_O +CH_3_-O-(CH_2_)_2_-OH → M_1_-OH+ M_2_-(CH_2_)_2_-O-CH_3_+NO↑+H_2_O↑ (3)

M_1_-(CH_2_)_2_-O-CH_3_+H_2_O+O_2_ → M_1_-OH+ CH_3_-O-(CH_2_)_2_-OH↑ (4)

M_1_-(CH_2_)_2_-O-CH_3_+ M_2_-OH+O_2_ → M_1_O_X_+ CH_3_-O-(CH_2_)_2_-OH↑ (5)

M_1_-OH+M_2_-OH → M_1_-OH-M_2_+H_2_O (6)

M_1_-OH-M_2_+M_1_-OH → M_1_-O-M_2_-M_1_+H_2_O (7)

M_1_-OH-M_2_+M_1_-OH → M_1_-O-M_2_-M_1_+H_2_O (8)

where M_1_ and M_2_ equal to metals.

The hydroxyl groups left the system in different forms or combined with metal atoms after transformation, resulting in a decrease in the hydroxyl group content in the system. As one of the electron conduction pathways [54,55], the reduction of the hydroxyl group content of hydroxyl group will contribute to a lower leakage current of the insulating layer in the MIM device.

According to the studies of Song. K et al. [9], the proportion of metal oxides was higher than that of metal hydroxides when the annealing temperature increased. The relative dielectric constant of yttria is bigger than that of yttrium hydroxide, so the increase of oxide content has a positive influence on the dielectric properties.

**Figure 8:**
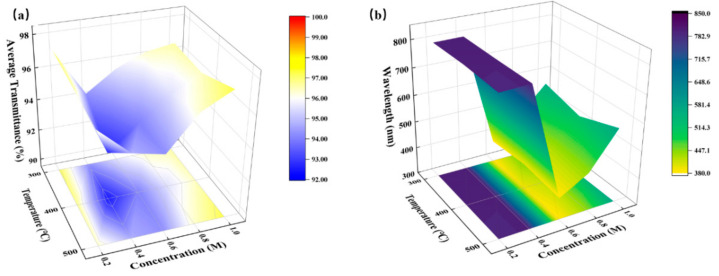
ZYAMO thin films with different concentrations after different temperature annealing. (**a**) Average transmittance in the visible region. (**b**) Maximal transmittance to wavelength in the visible region.

We characterized the optical properties of ZYAMO thin films with a UV-VIS spectrophotometer, and the results are displayed in Figure 8.

Combined with the film thickness results mentioned in Figure 4, it can be seen from Figure 8 that the larger the film thickness/roughness was, the greater the influence of atoms on light scattering and refraction would be. Thus, the transmittance would decrease as those parameters increase. Moreover, the peak level to the wavelength is different. Similar situations can be seen in previous studies [36]. In summary, the visible light transmittance of the samples under all conditions was more than 90%, and the value stayed at a high level as the concentration increased. Therefore, ZYAMO thin films have great potential in the preparation of transparent devices.

**Figure 9:**
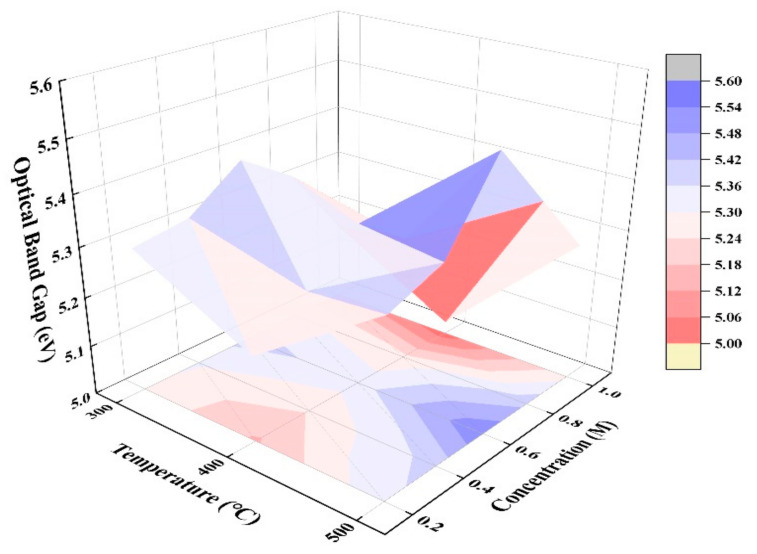
ZYAMO thin film optical band gap under different annealing temperatures and solution concentrations.

Absorption spectrums were also tested to calculate the absorptivity, and a linear fitting based on Formula (9) was made to figure out the optical band gap in the linear region [56]:

(9) $$\alpha h\nu =A{\left(h\nu -E_{g}\right)}^{1/2}$$

where *α* is the absorption coefficient, *hν* is the photon energy, *A* is a constant, and *E_g_* is the optical band gap. The calculated optical band gap values are shown in Figure 9. The optical band gap was the largest (~5.53 eV) at 500 °C and 0.6 M. Wang et al. [57] proved that the change of optical band gap of metal oxides was closely related to the density of the band tail states. The remaining organic matter and oxygen vacancy defects were also reasons of the disordered structure. These factors would cause the localization of electrons and holes at the bottom of the conduction band and the top of the valence band. The band tail states, and even the deep level states, were formed in this case, and the band gap narrowed [58]. Based on this, the trends were likely the result of the decrease of band tail states. When the annealing temperature went up, the oxygen defects reduced as the remaining organic matter decomposed. Moreover, the impact of the absorption and scattering of light were also weakened, so the transmittance increased when the annealing temperature was higher.

**Figure 10:**
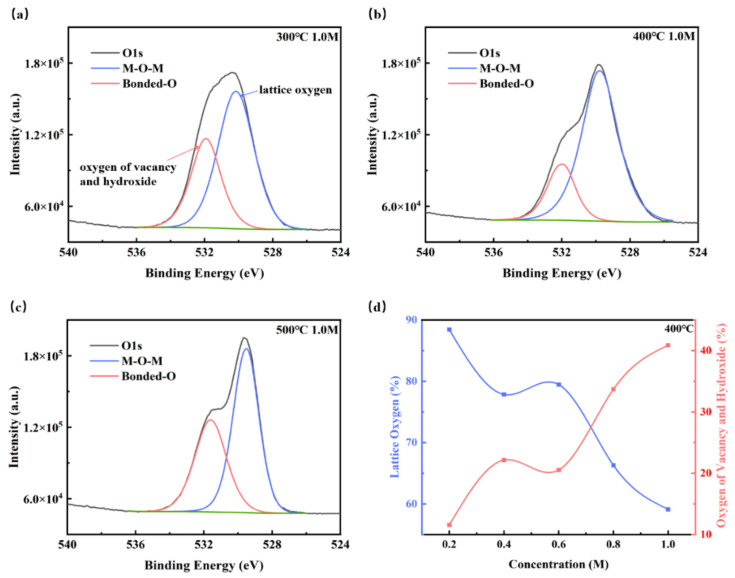
XPS spectra of oxygen 1 s for the ZYAMO thin films at: (**a**) 300 °C, 1.0 M, (**b**) 400 °C, 1.0 M, and (**c**) 500 °C, 1.0 M. (**d**) The tendency of oxygen content with concentration changes under 400 °C.

Since the oxygen vacancies and hydroxyl group have important effects on the electrical properties of the ZYAMO thin films, X-ray photoelectron spectra (XPS) were also used to clarify the chemical structures and compositions of the ZYAMO thin films, and the results are shown in Figure 10 and Table 1. It can be seen in Figure 10 that the oxygen peak position has a slight shift, which is likely to be affected by the electronegativity of the elements. Metals are less electronegative compared to hydrogen and oxygen. When the electronegativity increases, the electrons shift to the M/H side, reducing the electron density around O. Therefore, the oxygen nucleus will strengthen the bound of extranuclear electron, and the binding energy increases [59,60]. Figure 10a–c show the content of O1 (lattice oxygen) and O2 (oxygen of vacancy and hydroxide) change with temperature from 300 °C to 500 °C under 1.0 M. Using data from previous research [23,61,62], the three kinds of peak positions can be determined as ~530 eV, ~531 eV and ~532 eV, respectively. The peak position of O2 is closer to oxygen vacancy when the annealing temperature is 300 °C and 500 °C. Most of the oxygen came from -OH under the condition of 400 °C, though the proportion of O2 decreases. This implies that it is more likely to obtain thin films with a higher relative dielectric constant under 400 °C. Similar results can be found in the study of Liang et al. [24]. We speculated that the content of bound oxygen dropped as the temperature climbed, which is easier for solvent to decompose, but the oxygen defects increased as well after 400 °C. From Figure 10d, it can be observed that the content of O2 increased as the concentration went up. Moreover, it can be seen that the percentage of O1 (lattice oxygen) decreased when the concentration increased from Table 1. There are more oxygen defects when the concentration is higher because of a higher disordered structure. The above results indicate that higher entropy of the zirconium-yttrium-aluminum-magnesium-oxide system has partly influenced the electrical properties.

### 3.2. Electrical Properties

**Figure 11:**
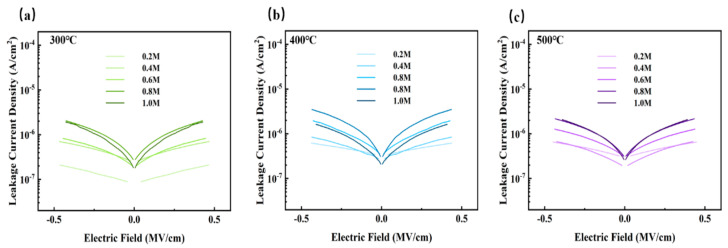
ZYAMO thin film leakage current density after different temperature annealing: (**a**) 300 °C, (**b**) 400 °C, and (**c**) 500 °C.

**Figure 12:**
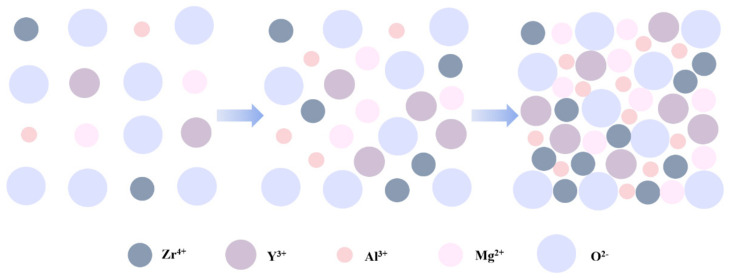
Sketch map of variations in the atomic arrangement as the concentration increases.

**Figure 13:**
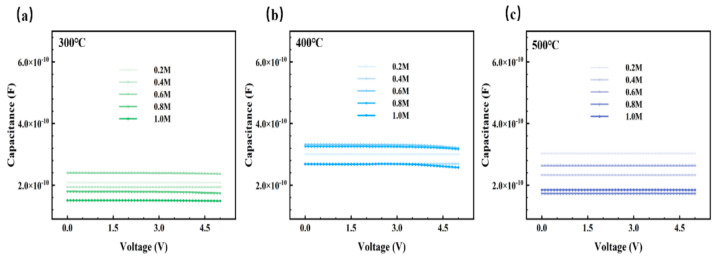
ZYAMO thin film capacitance after different temperature annealing: (**a**) 300 °C, (**b**) 400 °C, and (**c**) 500 °C.

In order to characterize the electrical properties, we prepared metal-insulator-metal (MIM) device. Based on this, a semiconductor analyzer was used to test the electrical characteristics of the MIM device, and the I-V and C-V results are displayed in Figure 11, Figure 12 and Figure 13. The most popular mechanisms of explanations of insulator conduction are Schottky emission and Poole–Frenkel emission [63]. Schottky emission conduction is a process that occurs at the interface when a metal contacts with an insulating layer, and the formed barrier will block the transmission of carriers. This phenomenon is more likely to happen in the insulators with less defects [64]. Poole–Frenkel emission is closely related to the density of defect states and the thermal excitation of carriers. The rough surface is conducive to the generation of electric field [65], which leads to the enhancement of the Poole-Frenkel effect, forcing the emission of carriers caught in the trap states, and the leakage current increases as a result.

In summary, the leakage current is affected by many factors [31,55,65,66], such as microstructure, grain size, crystallization, surface roughness, defect density, etc. Among the results of the three groups, shown in Figure 11, leakage current of the films annealed at 400 °C was relatively higher due to the growing conductive pathways of electrons caused by a more obvious crystallization of metal oxides when the temperature increased from 300 °C to 400 °C, and it presented an upward trend as the concentration climbed. More than that, a higher leakage current in the 400 °C group was also a result of larger band gaps, providing favorable conditions for space charges to convey [67]. However, the current decreased when the temperature increased to 500 °C, possibly because there were fewer defects [68]. Lower oxygen defects limit the conductivity when the factors of defects account for the main position at a higher temperature. Based on the tests and results mentioned above, ZYAMO dielectric films can reach a lower leakage current of less than 3.5 × 10^−6^ A/cm^2^. From Figure 12, the possible reason of the improved dielectric properties is the highly disordered structure of the system.

According to the C-V results in Figure 13, the middle temperature group showed a stronger capacity of charge storage, and the capacitance of this group under different concentration had less variation compared with that of 300 °C and 500 °C. The fluctuation in curves might be caused by a greater effect of trapping/detrapping of electrons [69].

**Figure 14:**
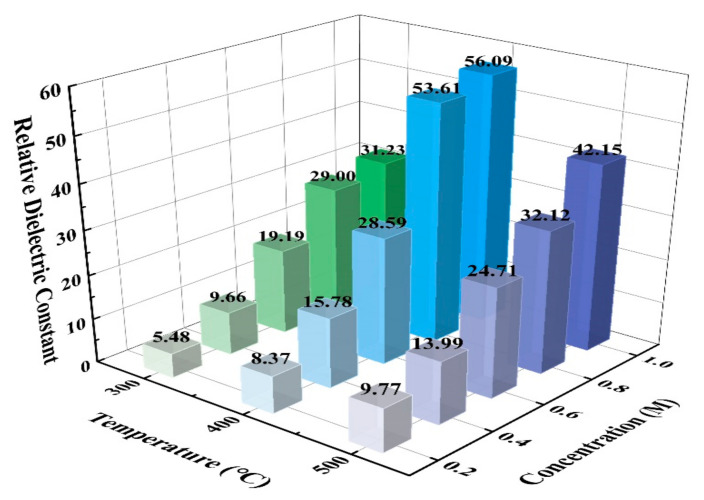
ZYAMO thin film relative dielectric constants under different annealing temperatures and solution concentrations.

The relative dielectric constant can be calculated by formula (10), where *C* is capacitance, k is the relative dielectric constant, *ε_0_* is the dielectric constant of vacuum (8.85 × 10^−12^ F/m), *S* is the area of the electrodes, *d* is the thickness of dielectric layer, and the results are shown in Figure 14.

(10) $$C=k\varepsilon_{0}\frac{S}{d}$$

The relative dielectric constant increased as the concentration increased, and it reached the maximal values under the condition of 400 °C. Therefore, it is important to control the annealing temperature, which influenced the degree of crystallization [66]. Moreover, the proportion of oxides was another factor that had an effect on the relative dielectric constant [9,33,70]. Compared to the binary metal films in previous studies, which reached limited relative dielectric constants [24,25,26], ZYAMO dielectric films applied in this study made a breakthrough for the dielectric constant. The carrier was hindered and accumulated by various cationic complex arrangements and the states of electrons had been influenced by disordered structure, so there was a greater possibility to store charges [71,72]. In summary, the increase of metal cationic and oxides help to improve the dielectric properties apart from the superiorities provided by various metal elements.

## 4. Conclusions

In this study, we fabricated ZYAMO thin films by using the solution method to achieve a high relative dielectric constant and a large optical band gap. The surface characteristics, optical properties, and electrical properties of the dielectric film were measured through various tests. From the results, it can be seen that the optical band gaps are wider when the concentration is 0.6 M and the annealing temperature is 500 °C. Moreover, the leakage current density is lower and the relative dielectric constants are higher under the condition of 400 °C. To summarize, ZYAMO thin films applied in dielectric layer showed the best performance at 1.0 M and 400 °C, and it reached a relatively wide band gap of 5.03 eV, a high relative dielectric constant of 56.09 and a relatively low leakage current density of 1.63 × 10^−6^ A/cm^2^@ 0.5 MV/cm. Consequently, the significant development also offered a foundation for further study in high entropy metal oxide films.

## Figures and Tables

**Table 1:** The relative proportion of lattice oxygen under different conditions.

Concentration(M)	Lattice Oxygen at 300 °C (%)	Lattice Oxygen at 500 °C (%)
0.2	60.38	88.43
0.4	56.63	77.85
0.6	52.45	79.46
0.8	41.94	66.30
1.0	64.23	59.12

## Data Availability

Data are contained within the article.

## References

[1] Sun Y.H., Kim J., Chatterjee N., Swisher S.L. (2021). Investigation of the Determining Factors for the "Mobility Boost" in High-k-Gated Transparent Oxide Semiconductor Thin-Film Transistors. Adv. Electron. Mater..

[2] Luo C.L., Huang T., Li C.H., Zhang Y., Zou Z.M., Li Y.S., Tao R.Q., Gao J.W., Zhou G.F., Lu X.B. (2021). Enhancement of electrical properties of solution-processed oxide thin film transistors using ZrO_2_ gate dielectrics deposited by an oxygen-doped solution. J. Phys. D Appl. Phys..

[3] Kim J., Choi S., Jo J.W., Park S.K., Kim Y.H. (2018). Solution-processed lanthanum-doped Al_2_O_3_ gate dielectrics for high-mobility metal-oxide thin-film transistors. Thin Solid Film..

[4] Xu F., Liu A., Liu G.X., Shin B., Shan F.K. (2015). Solution-processed yttrium oxide dielectric for high-performance IZO thin-film transistors. Ceram. Int..

[5] Liu X.Q., Liu W., Xiao X.H., Wang C.L., Fan Z.Y., Qu Y.Q., Cai B., Guo S.S., Li J.C., Jiang C.Z. (2013). High performance amorphous ZnMgO/carbon nanotube composite thin-film transistors with a tunable threshold voltage. Nanoscale.

[6] Byun H.-R., You E.-A., Ha Y.-G. (2019). Room-temperature solution-processed, ZrO_x_-based hybrid gate dielectrics for low-voltage organic thin-film transistors on plastic substrates. Appl. Phys. Lett..

[7] Fan C., Liu A., Meng Y., Guo Z., Liu G., Shan F. (2017). Solution-Processed SrOₓ-Gated Oxide Thin-Film Transistors and Inverters. IEEE Trans. Electron. Devices.

[8] Son B.G., Je S.Y., Kim H.J., Lee C.K., Lee C.K., Hwang A.Y., Won J.Y., Song J.H., Choi R., Jeong J.K. (2013). High-performance In-Zn-O thin-film transistors with a soluble processed ZrO_2_ gate insulator. Phys. Status Solidi Rapid Res. Lett..

[9] Song K., Yang W., Jung Y., Jeong S., Moon J. (2012). A solution-processed yttrium oxide gate insulator for high-performance all-solution-processed fully transparent thin film transistors. J. Mater. Chem..

[10] Adamopoulos G., Thomas S., Wöbkenberg P.H., Bradley D.D.C., McLachlan M.A., Anthopoulos T.D. (2011). High-Mobility Low-Voltage ZnO and Li-Doped ZnO Transistors Based on ZrO_2_ High-k Dielectric Grown by Spray Pyrolysis in Ambient Air. Adv. Mater..

[11] Lan L.F., Zhang P., Peng J.B. (2016). Research progress on oxide-based thin film transisitors. Acta Phys. Sin..

[12] Park J.S., Jeong J.K., Mo Y.G., Kim S. (2009). Impact of high-k TiOx dielectric on device performance of indium-gallium-zinc oxide transistors. Appl. Phys. Lett..

[13] Nomura K., Ohta H., Ueda K., Kamiya T., Hirano M., Hosono H. (2003). Thin-film transistor fabricated in single-crystalline transparent oxide semiconductor. Science.

[14] Zhong Y.X., Xie Y., Zhou S.X., Yuan W.J., Shi M.Y., Yao R., Xu M., Wang L., Lan L., Peng J.B. (2017). Oxide semiconductor thin film transistor device print fabrication based on solution method. Chin. J. Liq. Cryst. Disp..

[15] Zhang X., Wang S., Yao R., Liu X., Hou D., Ye Q., Li J., Huang J., Cao X., Peng J. (2020). Preparation and optimization of SnO_x_ thin film by solution method at low temperature. Superlattices Microstruct..

[16] Zhou S., Zhang J., Fang Z., Ning H., Cai W., Zhu Z., Liang Z., Yao R., Guo D., Peng J. (2019). Thermal effect of annealing-temperature on solution-processed high-k ZrO_2_ dielectrics. RSC Adv..

[17] Zhou S., Cai W., Zhennan Z., Tao R., Yao R., Wang Y., Fang Z., Zhou Z., Peng J. (2019). Fabrication of High-Performance Solution Processed Thin Film Transistors by Introducing a Buffer Layer. Appl. Surf. Sci..

[18] Cai W., Ning H., Zhou S., Zhu Z., Yao R., Chen J., Tao R., Fang Z., Lu X., Peng J. (2019). Effective Evaluation Strategy Toward Low Temperature Solution-Processed Oxide Dielectrics for TFT Device. IEEE J. Electron Devices Soc..

[19] Kang I., Avis C., Kang D.H., Jang J. (2011). Low-Voltage Poly-Si TFTs with Solution-Processed Aluminum Oxide Gate Dielectric. Electrochem. Solid State Lett..

[20] Avis C., Jang J. (2011). High-performance solution processed oxide TFT with aluminum oxide gate dielectric fabricated by a sol-gel method. J. Mater. Chem..

[21] Chun Y.S., Chang S., Sang Y.L. (2011). Effects of gate insulators on the performance of a-IGZO TFT fabricated at room-temperature. Microelectron. Eng..

[22] Liu A., Liu G.X., Zhu H.H., Meng Y., Song H.J., Shin B., Fortunato E., Martins R., Shan F. (2015). A water-induced high-k yttrium oxide dielectric for fully-solution-processed oxide thin-film transistors. Curr. Appl. Phys..

[23] Yang W., Song K., Jung Y., Jeong S., Moon J. (2013). Solution-deposited Zr-doped AlOx gate dielectrics enabling high-performance flexible transparent thin film transistors. J. Mater. Chem. C.

[24] Liang Z., Zhou S., Cai W., Fu X., Ning H., Chen J., Yuan W., Zhu Z., Yao R., Peng J. (2020). Zirconium-Aluminum-Oxide Dielectric Layer with High Dielectric and Relatively Low Leakage Prepared by Spin-Coating and the Application in Thin-Film Transistor. Coatings.

[25] Wu W., Javaid K., Liang L., Yu J., Liang Y., Song A., Yao M., Lan L., Cao H. (2018). Aqueous Solution Induced High-Dielectric-Constant AlO_x_:Y Films for Thin-Film Transistor Applications. J. Nanosci. Nanotechnol..

[26] Woods K.N., Chiang T.H., Plassmeyer P.N., Kast M.G., Lygo A.C., Grealish A.K., Boettcher S.W., Page C.J. (2017). High-kappa Lanthanum Zirconium Oxide Thin Film Dielectrics from Aqueous Solution Precursors. ACS Appl. Mater. Interfaces.

[27] Wu H., Liang J., Jin G., Lao Y., Xu T. (2007). Transparent Thin-Film Transistors Using ZnMgO as Dielectrics and Channel. IEEE Trans. Electron Devices.

[28] Anderson J.T., Munsee C.L., Hung C.M., Phung T.M., Herman G.S., Johnson D.C., Wager J.F., Keszler D.A. (2007). Solution-processed HafSO_x_ and ZircSO_x_ inorganic thin-film dielectrics and nanolaminates. Adv. Funct. Mater..

[29] Zhong Y., Zhou S., Yao R., Wei C., Peng J. (2018). Fabrication of Zirconia Dielectric Layer by Spin Coating and Its Application in Thin Film Transistor. Chin. J. Lumin..

[30] Wilk G.D., Wallace R.M., Anthony J.M. (2001). High-κ gate dielectrics: Current status and materials properties considerations. J. Appl. Phys..

[31] Xu W., Wang H., Ye L., Xu J. (2014). The role of solution-processed high-κ gate dielectrics in electrical performance of oxide thin-film transistors. J. Mater. Chem. C.

[32] Ye P.D., Wilk G.D., Yang B., Kwo J., Gossmann H., Frei M., Mannaerts J.P., Sergent M., Hong M., Ng K.K. (2004). GaAs-based metal-oxide semiconductor field-effect transistors with Al_2_O_3_ gate dielectrics grown by atomic layer deposition. J. Electron. Mater..

[33] Chen W.Y., Jeng J.S., Chen J.S. (2012). Improvement of Mobility in ZnO Thin Film Transistor with an Oxygen Enriched MgO Gate Dielectric. ECS Solid State Lett..

[34] Ku C.J., Duan Z., Reyes P.I., Lu Y., Garfunkel E. (2015). Effects of Mg on the electrical characteristics and thermal stability of Mg_x_Zn_1-x_O thin film transistors. Appl. Phys. Lett..

[35] Umezawa N., Sato M., Shiraishi K. (2008). Reduction in charged defects associated with oxygen vacancies in hafnia by magnesium incorporation: First-principles study. Appl. Phys. Lett..

[36] Tsay C., Cheng C., Wang Y. (2012). Properties of transparent yttrium oxide dielectric films prepared by sol-gel process. Ceram. Int..

[37] Ting C.C., Fan H.Y., Tsai M.K., Li W.Y., Yong H.E., Lin Y.F. (2014). Improvement of electrical characteristics in the solution-processed nanocrystalline indium oxide thin-film transistors depending on yttrium doping concentration. Phys. Status Solidi A.

[38] Li H., Zhou Y., Liang Z., Ning H., Fu X., Xu Z., Qiu T., Xu W., Yao R., Peng J. (2021). High-Entropy Oxides: Advanced Research on Electrical Properties. Coatings.

[39] McNaught A., Wilkinson A. (1997). IUPAC Compendium of Chemical Terminology.

[40] Yao H., Yuan Z., Xiong Z., Zhai Y., Li D. (2016). Microstructure and photoluminescence of SnO_2_ thin films prepared by reactive magnetron sputtering. Cailiao Daobao Mater. Rev..

[41] Hong Y., Zhanwen Z., Yong H., Sai L., Bo L. (2011). Factors influencing surface roughness of polyimide film. High Power Laser Part Beams.

[42] Singh G.V.P.B., Sonat C., Yang E.H., Unluer C. (2020). Performance of MgO and MgO-SiO_2_ systems containing seeds under different curing conditions. Cem. Concr. Compos..

[43] Zhang J., Fu X., Zhou S., Ning H., Wang Y., Guo D., Cai W., Liang Z., Yao R., Peng J. (2019). The Effect of Zirconium Doping on Solution-Processed Indium Oxide Thin Films Measured by a Novel Nondestructive Testing Method (Microwave Photoconductivity Decay). Coatings.

[44] Aphane M.E., van der Merwe E.M., Strydom C.A. (2009). Influence of hydration time on the hydration of MgO in water and in a magnesium acetate solution. J. Therm. Anal. Calorim..

[45] Khanra A.K. (2007). Reaction chemistry during self-propagating high-temperature synthesis (SHS) of H_3_BO_3_-ZrO_2_-Mg system. Mater. Res. Bull..

[46] Esparza A.A., Ferguson R.E., Choudhuri A., Love N.D., Shafirovich E. (2018). Thermoanalytical studies on the thermal and catalytic decomposition of aqueous hydroxylammonium nitrate solution. Combust. Flame.

[47] Tang C.-W., Wang C.-B., Chien S.-H. (2008). Characterization of cobalt oxides studied by FT-IR, Raman, TPR and TG-MS. Thermochim. Acta.

[48] Kakade M.B., Ramanathan S., Ravindran P.V. (2003). Yttrium aluminum garnet powders by nitrate decomposition and nitrate–urea solution combustion reactions—a comparative study. J. Alloy. Compd..

[49] Xu W., Wang H., Xie F., Chen J., Cao H., Xu J.-B. (2015). Facile and Environmentally Friendly Solution-Processed Aluminum Oxide Dielectric for Low-Temperature, High-Performance Oxide Thin-Film Transistors. ACS Appl. Mater. Interfaces.

[50] Xu W., Long M., Zhang T., Liang L., Cao H., Zhu D., Xu J.-B. (2017). Fully solution-processed metal oxide thin-film transistors via a low-temperature aqueous route. Ceram. Int..

[51] Xu Z., Xian-zhe L.I.U., Wei-jian Y., Yu-xi D., Xiao-chen Z., Shuang W., Jia-liang W., Yao R., Jun-biao P. (2019). Effect of Annealing Temperature on Properties of SnO_2_ Thin Films Prepared by Spin Coating. Chin. J. Lumin..

[52] Liu G.X., Liu A., Zhu H.H., Shin B., Fortunato E., Martins R., Wang Y.Q., Shan F.K. (2015). Low-Temperature, Nontoxic Water-Induced Metal-Oxide Thin Films and Their Application in Thin-Film Transistors. Adv. Funct. Mater..

[53] Hwan Hwang Y., Seo J.-S., Moon Yun J., Park H., Yang S., Ko Park S.-H., Bae B.-S. (2013). An ‘aqueous route’ for the fabrication of low-temperature-processable oxide flexible transparent thin-film transistors on plastic substrates. NPG Asia Mater..

[54] Jeong S., Jeong Y., Moon J. (2008). Solution-Processed Zinc Tin Oxide Semiconductor for Thin-Film Transistors. J. Phys. Chem. C.

[55] Jeong S., Ha Y.-G., Moon J., Facchetti A., Marks T.J. (2010). Role of Gallium Doping in Dramatically Lowering Amorphous-Oxide Processing Temperatures for Solution-Derived Indium Zinc Oxide Thin-Film Transistors. Adv. Mater..

[56] Ratana T., Amornpitoksuk P., Ratana T., Suwanboon S. (2009). The wide band gap of highly oriented nanocrystalline Al doped ZnO thin films from sol-gel dip coating. J. Alloy. Compd..

[57] Wang J.P., Wang Z.Y., Huang B.B., Ma Y.D., Liu Y.Y., Qin X.Y., Zhang X.Y., Dai Y. (2012). Oxygen Vacancy Induced Band-Gap Narrowing and Enhanced Visible Light Photocatalytic Activity of ZnO. ACS Appl. Mater. Interfaces.

[58] Seo H., Park C.J., Cho Y.J., Kim Y.B., Choi D.K. (2010). Correlation of band edge native defect state evolution to bulk mobility changes in ZnO thin films. Appl. Phys. Lett..

[59] Lan L., Song W., Lin Z., Xiao P., Wang L., Ning H., Wang D., Peng J. (2015). Effects of Nd in Nd_x_In_1-x_O_3_ Semiconductors for Thin-Film Transistors. IEEE Trans. Electron. Devices.

[60] Jeong Y., Bae C., Kim D., Song K., Woo K., Shin H., Cao G., Moon J. (2010). Bias-Stress-Stable Solution-Processed Oxide Thin Film Transistors. ACS Appl. Mater. Interfaces.

[61] Jung Y., Yang W., Koo C.Y., Song K., Moon J. (2012). High performance and high stability low temperature aqueous solution-derived Li-Zr co-doped ZnO thin film transistors. J. Mater. Chem..

[62] Tigunta S., Sando D., Chanlek N., Supadee L., Pojprapai S. (2020). Effect of gas atmospheres on degradation of MgO thin film magnetic tunneling junctions by deionized water. Thin Solid Film..

[63] Sze S.M. (1981). Physics of Semiconductor Devices.

[64] Chakraborty S., Bera M.K., Bhattacharya S., Maiti C.K. (2005). Current conduction mechanism in TiO_2_ gate dielectrics. Microelectron. Eng..

[65] Zhao Y.P., Wang G.C., Lu T.M., Palasantzas G., De Hosson J.T.M. (1999). Surface-roughness effect on capacitance and leakage current of an insulating film. Phys. Rev. B.

[66] Hsu C.H., Yan S.F. (2011). Fabrication and Characterization of ZnNb_2_O_6_ Thin Films Using Sol-Gel Method. J. Am. Ceram. Soc..

[67] Campbell S.A., Kim H.S., Gilmer D.C., He B., Ma T., Gladfelter W.L. (1999). Titanium dioxide (TiO_2_)-based gate insulators. IBM J. Res. Dev..

[68] Robertson J. (2005). High dielectric constant gate oxides for metal oxide Si transistors. Rep. Prog. Phys..

[69] Tsui B.Y., Hsu H.H., Cheng C.H. (2010). High-Performance Metal-Insulator-Metal Capacitors With HfTiO/Y_2_O_3_ Stacked Dielectric. IEEE Electron. Device Lett..

[70] Lee J.H., Kim H.S., Kim S.H., Jang N.W., Yun Y. (2014). Characterization of magnesium oxide gate insulators grown using RF sputtering for ZnO thin-film transistors. Curr. Appl. Phys..

[71] Zhou S., Pu Y., Zhang Q., Shi R., Guo X., Wang W., Ji J., Wei T., Ouyang T. (2020). Microstructure and dielectric properties of high entropy Ba(Zr_0.2_Ti_0.2_Sn_0.2_Hf_0.2_Me_0.2_)O_3_ perovskite oxides. Ceram. Int..

[72] Zhao Y., Zhu J., Wang H., Ma Z., Gao L., Liu Y., Liu Y., Shu Y., He J. (2021). Enhanced optical reflectivity and electrical properties in perovskite functional ceramics by inhibiting oxygen vacancy formation. Ceram. Int..

